# The role of short-chain fatty acid in metabolic syndrome and its complications: focusing on immunity and inflammation

**DOI:** 10.3389/fimmu.2025.1519925

**Published:** 2025-02-07

**Authors:** Wenqian Yu, Siyuan Sun, Yutong Yan, Hong Zhou, Ziyi Liu, Qiang Fu

**Affiliations:** ^1^ Dongzhimen Hospital, Beijing University of Chinese Medicine, Beijing, China; ^2^ First Clinical Medical College, Beijing University of Chinese Medicine, Beijing, China

**Keywords:** short chain fatty acids, metabolic syndrome, inflammation, immunity, complications

## Abstract

Metabolic syndrome (Mets) is an important contributor to morbidity and mortality in cardiovascular, liver, neurological, and reproductive diseases. Short-chain fatty acid (SCFA), an organismal energy donor, has recently been demonstrated in an increasing number of studies to be an important molecule in ameliorating immuno-inflammation, an important causative factor of Mets, and to improve lipid distribution, blood glucose, and body weight levels in animal models of Mets. This study reviews recent research advances on SCFA in Mets from an immune-inflammatory perspective, including complications dominated by chronic inflammation, as well as the fact that these findings also contribute to the understanding of the specific mechanisms by which gut flora metabolites contribute to metabolic processes in humans. This review proposes an emerging role for SCFA in the inflammatory Mets, followed by the identification of major ambiguities to further understand the anti-inflammatory potential of this substance in Mets. In addition, this study proposes novel strategies to modulate SCFA for the treatment of Mets that may help to mitigate the prognosis of Mets and its complications.

## 1 Introduction

Metabolic syndrome (Mets) is a group of clinical syndromes including abdominal obesity, hypertension, hyperlipidemia, hyperglycemia, and a series of risk factors for cardiovascular and cerebrovascular diseases, and its core mechanisms are disorders of glucose and lipid metabolism and insulin resistance. As a result of a complex interplay of genetic and lifestyle factors (1), mostly increased consumption of high-calorie, low-fiber fast food and reduced physical activity due to longer commutes and sedentary work–life habits, it is now a truly global problem, with prevalence rates in urban populations in some developing countries often higher than in Western countries, and with global prevalence rates estimated to be approximately a quarter of the world’s population (2). Previous studies have shown that Mets is an important risk factor for cerebrovascular diseases and chronic kidney diseases (3, 4). The study of Mets is important for human health issues.

In recent years, the role of intestinal flora in the human body has received more attention, and more research on SCFA has been conducted. Studies on the application of SCFA in Mets have gradually begun to appear in the public’s eye, and they have been found to improve the distribution of lipids, glucose, and body weight levels in animal models of Mets (5, 6). It is well known that Mets is a systemic chronic inflammatory disease, and SCFA can regulate immunity and inflammation, and the correlation between immunity and Mets has received more attention (7). However, the specific mechanisms are still unclear, and there is no summary report of anti-inflammatory and anti-immune mechanisms in the various complications of Mets. Recent advances in the regulation of immunity and inflammation by SCFA provide new insights into inflammation-related Mets. This review will discuss the effector pathways associated with SCFA in inflammatory Mets and its complications, the mechanisms by which SCFA ameliorates inflammation in Mets, and the treatment of the components of inflammatory Mets and its complications with SCFA.

## 2 Search strategy

This review followed the PRISMA guidelines. The primary search for article screening used in this review was conducted using PubMed (1,021), Web of Science (985), and Scopus (128) and the medical subject headings (short-chain fatty acid, inflammation, and metabolic syndrome). Using the PubMed database as an example, we present our search strategy: ((((((((((((Short Chain Fatty Acid[MeSH Terms]) OR (Short-Chain Fatty Acid[Title/Abstract])) OR (Volatile Fatty Acid[Title/Abstract])) AND (Inflammation[MeSH Terms])) OR (Innate Inflammatory Response[Title/Abstract])) AND (Metabolic Syndrome[MeSH Terms])) OR (Reaven Syndrome X[Title/Abstract])) OR (Metabolic Syndrome X[Title/Abstract])) OR (Insulin Resistance Syndrome X[Title/Abstract])) OR (Metabolic Cardiovascular Syndrome[Title/Abstract])) OR (Metabolic X Syndrome[Title/Abstract])) OR (Dysmetabolic Syndrome X[Title/Abstract])) OR (Cardiometabolic Syndrome[Title/Abstract]).

**Figure 1:**
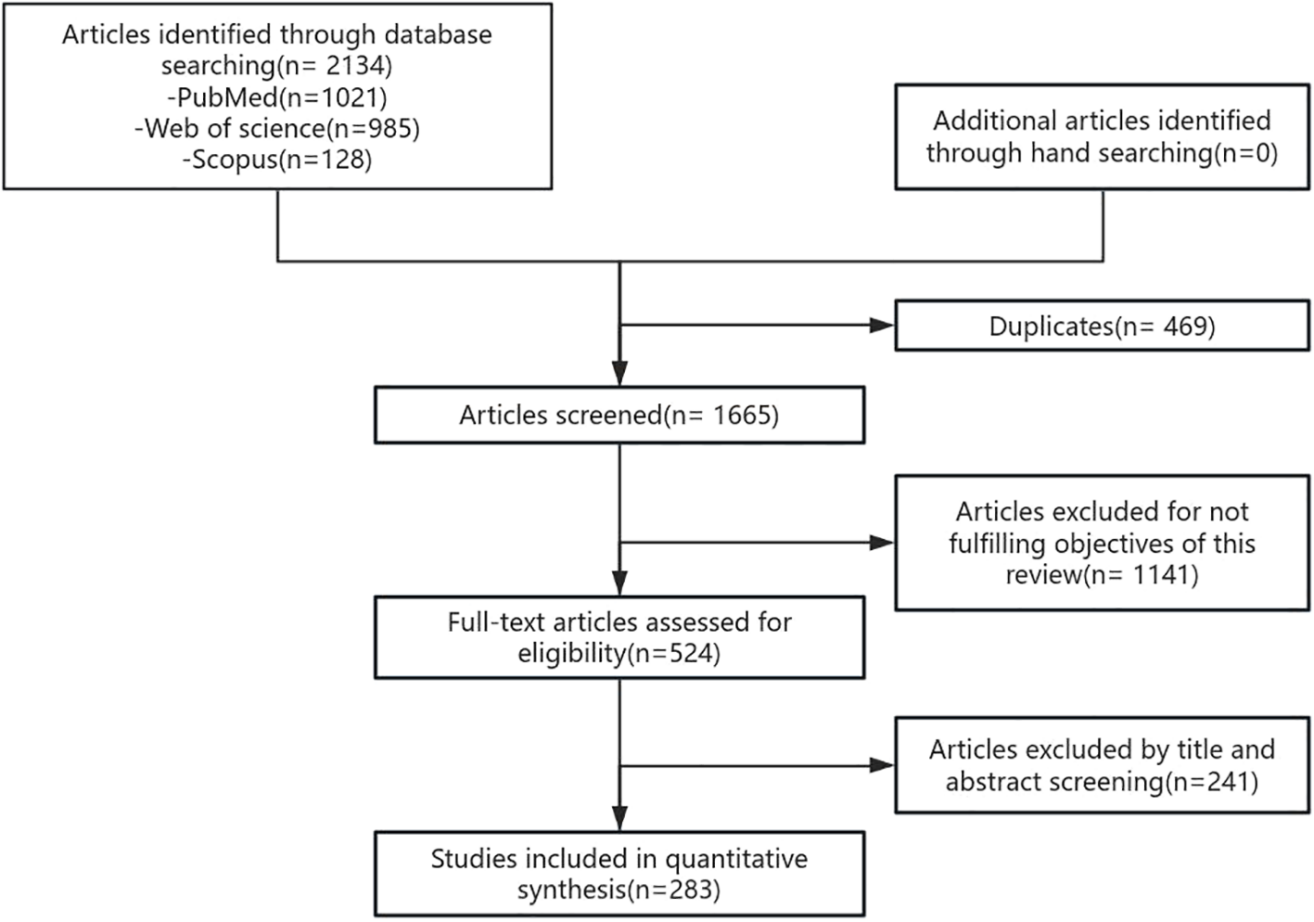
Search strategy. The flowchart outlines the search and screening process for the study. The search was first performed in PubMed, Web of Science, and Scopus, then duplicate articles and articles that did not meet the research criteria were removed, and finally relevant articles were selected for further research.

The literature screening was conducted collaboratively by two researchers. Following a rigorous selection process, 143 articles were included in our study. The PRISMA flowchart illustrates the process of identifying and screening articles related to the role of SCFA as an anti-inflammatory and anti-immune mechanism in the inflammatory metabolic syndrome (**Figure 1**).

## 3 Gut flora, inflammation, and Mets

**Figure 2:**
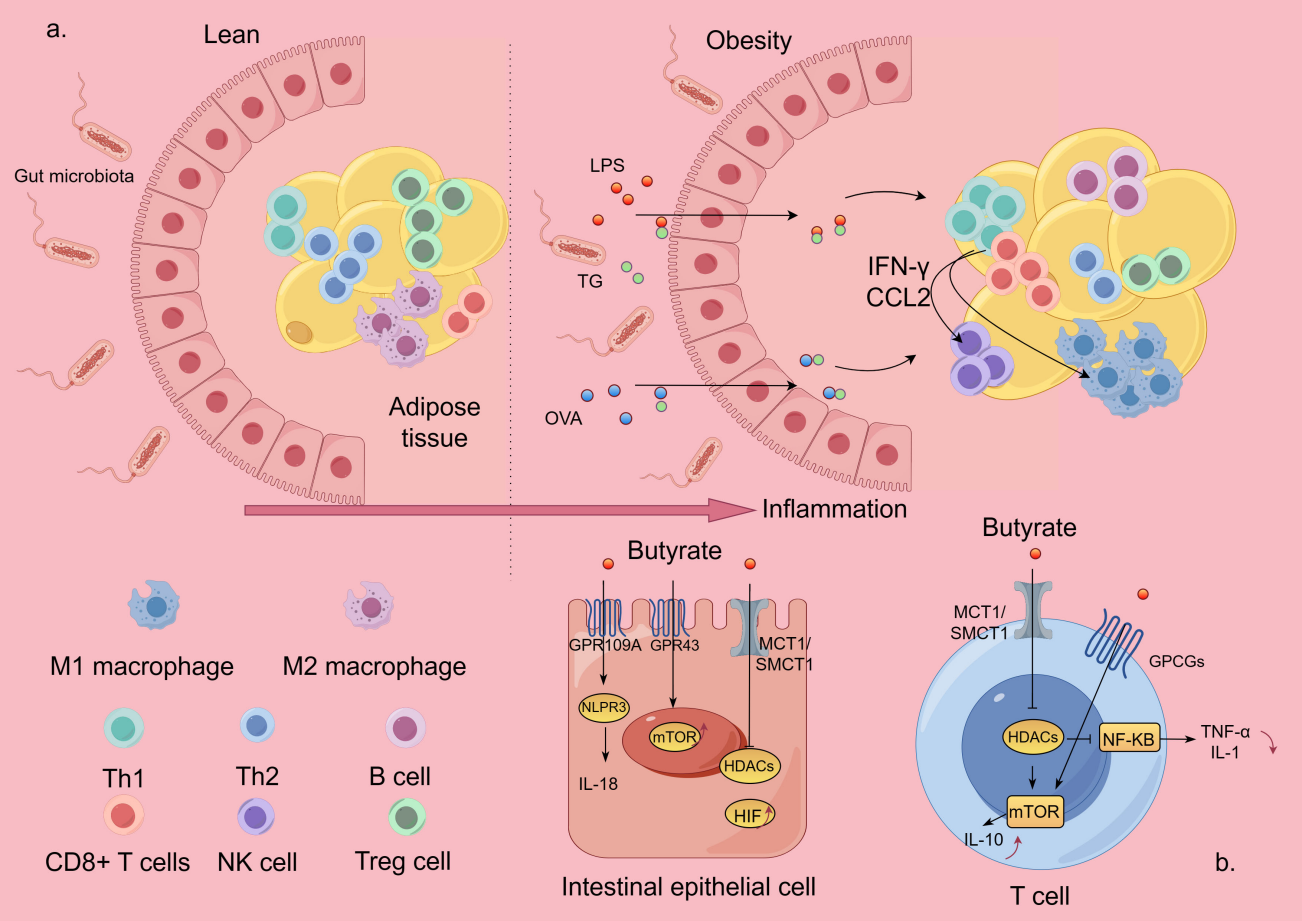
Inflammatory mechanisms of Mets and effector pathways of SCFA. The figure is drawn with Figdraw.com. **(A)** In Mets, hypertrophied adipose tissue disrupts intestinal barrier protection and promotes intestinal absorption of antigenic substances from the gut, which, in turn, induces an inflammatory immune response. **(B)** SCFA exerts its biological effects by inhibiting HDAC or activating GPCRs. LPS, lipopolysaccharides; OVA, ovalbumin; TG, triglycerides; NK, natural killer; TH, T-helper cell; Treg, T-regulatory cell; HDAC, histone deacetylase; GPCRs, G protein-coupled receptors.

High-fat diets and carbohydrate-rich diets are important triggers of Mets, which may weaken the adhesion of tight junction proteins in the gastrointestinal tract, resulting in leaky gut syndrome. Lipopolysaccharides (LPS) produced by harmful bacteria may infiltrate into the bloodstream through the portal vein, resulting in endotoxemia and inflammatory reactions, leading to inflammation of important organs related to metabolism, such as the liver and pancreas, as well as changes in the composition and number of intestinal flora. The metabolites produced by the altered intestinal microorganisms, such as SCFA and ethanol, may affect the bile acid and fat metabolism process in the liver, leading to the accumulation of fat, which, in turn, induces hepatic steatosis and triggers the development of Mets (8). In this process, immune imbalance plays an integral role in chronic inflammation, adipose tissue dysfunction, and gut flora disruption. Abdominal adipose tissue accumulation in Mets patients, especially visceral adipose tissue accumulation, exerts shear mechanical stress on the extracellular environment (9) and promotes intestinal uptake of antigenic substances therein, which, in turn, induces an inflammatory immune response, especially in tissues that are in close proximity to the intestinal tract, and this effect may be exacerbated by the abundance of lipids in a high-fat diet. Intestinal absorption of dietary fat promotes the uptake of LPS and protein antigens of intestinal bacterial origin (10, 11). Lack of immune tolerance to this antigen causes marked inflammatory responses in mesenteric adipose tissue, and a high-fat diet increases these inflammatory responses (12). Over time, these responses lead to reduced glucose tolerance. During diet-induced obesity, intestinal absorption of antigens is involved in T-cell activation and recruitment in visceral adipose tissue. The pro-inflammatory environment in visceral adipose may impair tolerance to these antigens by increasing free fatty acids and may lead to chronic inflammation. B lymphocytes are likewise recruited to adipose tissue after the onset of a high-fat diet, even before T cells (CD8+ T cells and TH1) are recruited (13). In the early stages of obesity, increased adipocytes release chemotactic adipokines and chemokines, such as CCL5, which help recruit pro-inflammatory cells of the adaptive immune system to adipose tissue (14). As obesity progresses, there is a progressive increase in the release of INF-γ and chemokines, such as CCL2, by CD8+ and TH1-type lymphocytes, which leads to the activation of NK cells and pro-inflammatory M1-type macrophages (e.g., **Figure 2A**). Activated M1-type macrophages infiltrate visceral adipose tissue and stimulate its release of large amounts of adipokines and chemotactic mediators such as tumor necrosis factor-α, interleukin-6, monocyte chemotactic protein-1, leptin, resistin, and vascular endothelial growth factor (15). Together, these factors induce a state of chronic, low-grade inflammation in the organism, which further contributes to insulin resistance and dyslipidemia, atherosclerosis, vascular dysfunction, and the development of nonalcoholic fatty liver disease (NAFLD) and Mets.

It can be seen that immune and inflammatory responses lead to intestinal bacterial imbalance, and intestinal bacterial imbalance is more likely to exacerbate the inflammatory response and promote Mets. Therefore, immunity, inflammation, and intestinal bacterial disorders often affect each other, forming a vicious cycle, but we can improve the intestinal internal environment, regulate the composition and abundance of SCFA, enhance the intestinal barrier function, reduce the leakage of LPS, and at the same time exert its anti-inflammatory effect, which, in turn, reduces the emergence of inflammation.

## 4 Causes and effector pathways of SCFA

### 4.1 Source

Since the origin of all things, microorganisms have coexisted with human beings, as if the microbial community has also played an important role in the evolutionary process of human beings, and all aspects of human biological functions are affected by them, among which, the gastrointestinal tract is the place where the greatest number and variety of commensal microorganisms are found in the human body, especially in the colon (16). Moreover, the total abundance of intestinal microorganisms accounted for more than 90% of the total intestinal microorganisms, such as the phylum Thick-walled Bacteria and the phylum Mycobacterium, which are the dominant bacterial flora involved in the production of SCFA in the intestinal tract. It has been demonstrated that SCFA, as an important metabolite of the intestinal bacterial flora, has a significant role in the body’s immunity, metabolism, endocrinology, and signaling, and that SCFAs are an important communication substance on the intestinal-organ axis (17–19). SCFA, also known as volatile fatty acid, is a general term for organic fatty acids containing two to six carbon atoms, mainly including acetic acid, propionic acid, and butyric acid, and consists of the intestinal metabolites of dietary fiber and protein (20, 21), of which dietary fiber is the main source of SCFA that can be divided into soluble (such as gelatin and inulin) and insoluble dietary fibers (such as various forms of resistant starch) (22).

SCFAs are produced by various prebiotics through fermentation processes such as Wood-Ljungdahl, carbon dioxide fixation, and acetyl-S coenzyme A condensation processes (23, 24). Prebiotics include antidigestive oligosaccharides (oligofructose), dietary fibers (e.g., inulin, pectin, and arabinoxylan), and resistant starches from various plant sources. Bacterial species that utilize prebiotics express carbohydrate-active enzymes (CAZymes) that degrade dietary fiber and thus produce SCFA (25). DF-rich diets increase the expression levels of microbiome-encoded CAZymes (26). Thus, the level of SCFA production in the colon is highly dependent on the microbial composition as well as the type and amount of DF in the diet. In addition to branched-chain amino acids such as valine, leucine, and isoleucine, amino acids can also be metabolized by microorganisms to produce acetic, propionic, and butyric acids (27). For example, threonine can be metabolized to the three main SCFAs. Microbes that efficiently produce SCFA are generally considered beneficial and are enriched in the intestines of healthy hosts with diets containing adequate levels of DF (28, 29). Previous studies have known that SCFAs, particularly butyric acid, can have anti-inflammatory effects on immune cell function. However, some researchers have found that the truth is not so absolute; propionic acid and butyric acid also have pro-inflammatory effects. Depending on the type and dose of SCFA used, these compounds may not impede the immune response, and in fact may even be stimulatory, with the exception of specific cell types (30). Cavaglieri et al. (31) demonstrated that butyric acid inhibited the production of IFN-γ by activated rat lymphocytes, whereas acetic and propionic acids increased this cytokine’s release. Specific mechanisms of effect are described below.

### 4.2 Effector pathways

Approximately 95% of SCFAs produced in the intestine are transported to intestinal epithelial cells by ion exchange, monocarboxylic acid transporter, or cell gap diffusion. Some of them are metabolized by intestinal cells to maintain intestinal homeostasis, while the other part is transported to various tissues and organs to play biological roles by inhibiting histone deacetylase (HDAC) or activating G protein-coupled receptors (GPCRs) (21) (e.g., **Figure 2B**) (32). The GPCRs such as GPR41, GPR43, and GPR109A have been found to be expressed on intestinal epithelial cells, adipose tissue, and immune cells including neutrophils, dendritic cells, macrophages, and lymphocytes. The expression of these GPCRs also varies in different tissues. A recent review has summarized the role of different receptors of SCFA in immune cells (33). Activation of these GPCRs can inhibit cAMP-dependent signaling pathways, activate the mTOR signaling pathway, and inhibit the NF-κB signaling pathway to reduce the inflammatory response (34, 35). NF-κB belongs to a family of nuclear transcription factors, including p50, p52, REL, REL-a, and REL-B, which is the primary response to noxious cellular stimuli. Several downstream mediators of the NF-κB pathway have been identified: tumor necrosis factor-α (TNF-α), interleukin (IL)-1, IL-2, IL-6, IL-8, IL-12, iNOS, COX2, chemokines, adhesion molecules, and colony-stimulating factors (36–38).

GPR41 is present in a wide range of tissues, whereas GPR43 is mainly expressed in lymphoid tissues and a variety of immune cells. Both GPR41 and GPR43 bind acetic, propionic, and butyric acids, whereas GPR109A is predominantly activated by butyric acid. The activation of GPR43 by SCFA can produce different effects according to different cell types. For example, it induces Nod-like receptor protein 3 (NLRP3) inflammatory vesicle activation and IL-18 secretion in colonic epithelial cells, promotes neutrophil recruitment to sites of inflammation, and enhances the differentiation and suppressive function of FOXP3+ regulatory T (regulatory T, Treg) cells (39). SCFAs (butyric and propionic acids) also exert their ability to modulate inflammatory and immune responses by inhibiting HDAC activity. When SCFAs enter cells by passive diffusion or through transmembrane vectors (e.g., SMCT1 and MCT1), they can directly bind to intracellular HDAC and inhibit its activity, which, in turn, inhibits the activation of the nuclear factor-κB (NF-κB). Emerging evidence suggests that butyric acid enhances p65 acetylation by inhibiting HDAC3 and HDAC6, also altering the lysine acetylation of non-histone proteins such as NF-κB subunit p65, leading to differential recruitment of NF-κB to pro-inflammatory gene promoters *in vitro* and *in vivo (* 40). In addition, SCFAs, particularly butyric acid, have recently been found to be activators of intracellular receptors that control immune responses, such as peroxisome proliferator-activated receptor-γ (PPARγ) (41) and the aromatic hydrocarbon receptor (42). Additionally, butyric acid can provide acetyl groups for histone acetylation (43). Butyrate affects gene expression in intestinal epithelial cells, macrophages, dendritic cells, and lymphocytes (especially Treg cells) by increasing histone acetylation and increasing the amount of open chromatin (44–46). Studies have observed that the effects of butyrate on immune and nonimmune cells result in alterations in pathways related to the cell cycle, cellular differentiation, antimicrobial responses, inflammation, fatty acid metabolism, and oxidative stress-related alterations in various pathways (46–48).

Overall, the diverse molecular targets of SCFAs illustrate that different SCFAs in different tissues can target immune and non-immune cells, modulate immunity in the gut and distal organs, and potentially protect against immune-mediated diseases.

## 5 Chronic inflammation due to gut microbial disorders contributes to Mets

**Figure 3:**
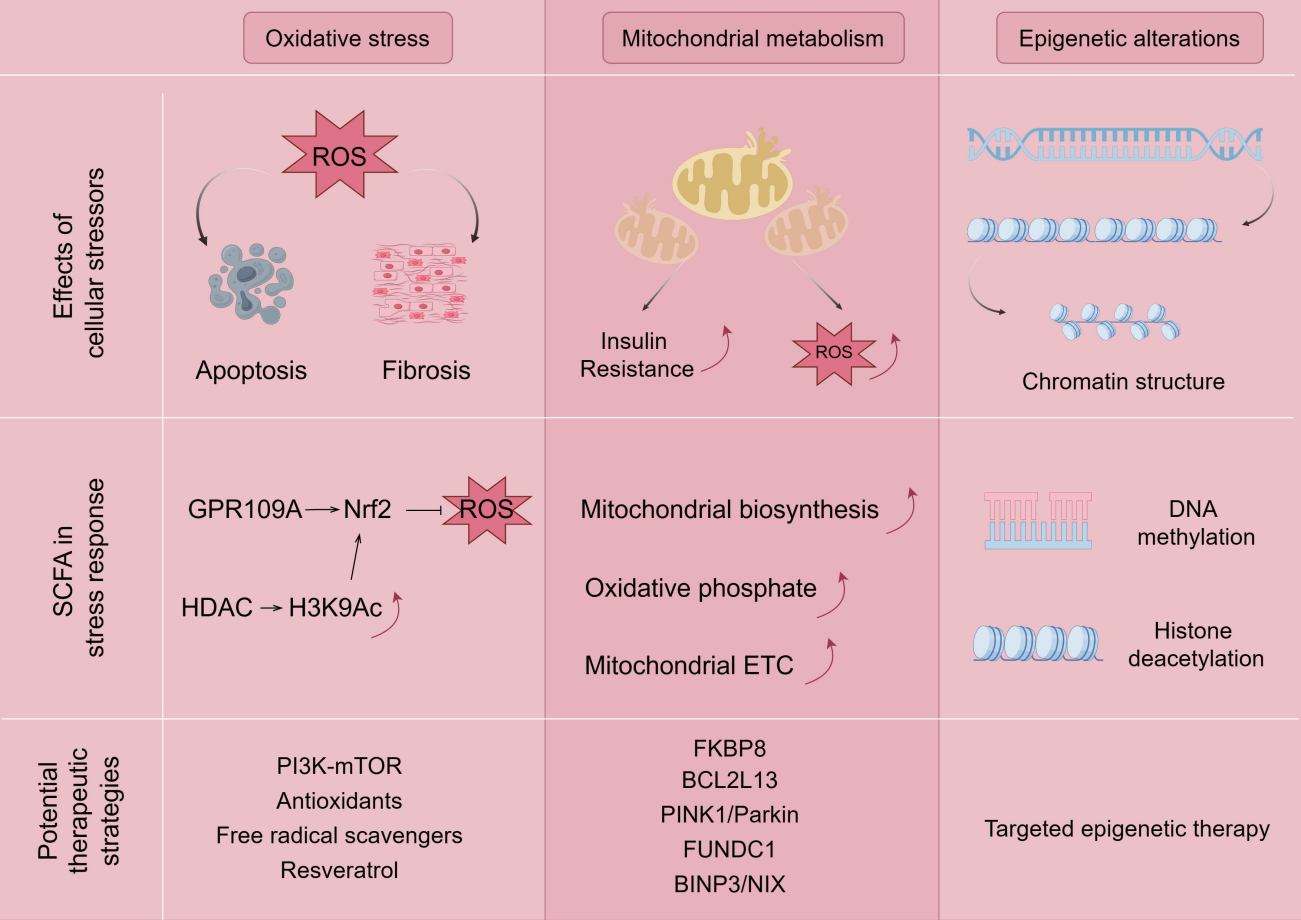
Mechanisms associated with SCFA ameliorating Mets by modulating oxidative stress, mitochondrial dysfunction, and epigenetic alterations. The figure is drawn with Figdraw.com. ROS, reactive oxygen species.

Gut microbes play a key role in maintaining physiological functions as they regulate host nutrition, energy harvesting, epithelial homeostasis, immune system, and drug metabolism while maintaining homeostasis (49). If the gut microbes are disturbed, the pre-existing homeostasis in the organism is disrupted and oxidative stress, mitochondrial dysfunction, epigenetic alterations, and, ultimately, Mets, occur (e.g., **Figure 3**).

### 5.1 Oxidative stress

Reactive oxygen species (ROS), including hydroxyl radicals, superoxide anion, and hydrogen peroxide, which are formed during the one-electron reduction of molecular oxygen, regulate a number of signaling pathways in the intestine and are considered to be central regulators of intestinal stem cell functions (50). Overproduction of ROS can occur in obesity, insulin resistance, hyperglycemia, chronic inflammation, dyslipidemia, and other pathologic diseases (51). Many studies have found that patients with Mets have lower plasma antioxidant enzyme activity and more biomarkers of oxidative damage compared to healthy individuals, which may contribute to oxidative stress (52). The overproduction of ROS leads to an oxidative stress environment, which also disrupts redox signaling and control, leading to an increase in growth factors and stress response components and activation of apoptotic pathways (53, 54). Disrupted redox signaling also promotes pro-inflammatory and pro-fibrotic pathways, affecting insulin metabolic signaling and endothelial dysfunction, and promoting cardiovascular and renal inflammation and fibrosis (54, 55), which can lead to target organ damage and the emergence of major components of Mets, such as hyperglycemia and hypoglycemia.

The direct and indirect mechanisms by which SCFAs regulate oxidative stress are through activation of the Keap1-Nrf2 cell signaling pathway (56, 57). In terms of the direct mechanism, SCFAs bind to the GPCR receptor to induce the direct activation of nuclear factor Nrf2 (58), and butyrate induces the activation of nuclear factor Nrf2 by recognizing the GPR109A receptor, which encodes an antioxidant enzyme that inactivates ROS (59). On the other hand, butyrate has a synergistic effect on Nrf2 activation because it diffuses into the cell lumen and indirectly activates Nrf2-dependent gene translocation and transcription by inhibiting HDAC and increasing the production of histone H3K9ac, which induces epigenetic modification of the Nrf2 promoter (56, 57, 60, 61). However, different types of SCFAs have different effects on ROS, and it has been shown that butyrate, propionate, and acetate treatments have reduced, no, or increased effects on ROS production in rat neutrophils (62). Among them, for acetate, which promotes the release of ROS from mouse neutrophils through the activation of GPR43 (63), the more plausible explanation for this phenomenon is the view of some researchers in the field of immunology, who believe that SCFAs may modulate inflammatory diseases by accelerating pathogen clearance through the activation of ROS (24). In fact, butyric acid does induce apoptosis or inhibit cell proliferation by increasing ROS levels, which, in turn, inhibits cancer progression (64, 65). The exact mechanism of SCFA and ROS needs to be continued to be explored by researchers.

Deletion of HDAC Sirtuin-1, an important target of SCFA, affects Mets as a result of activation of the phosphatidylinositol 3 kinase (PI3K)–mammalian target of rapamycin (mTOR) signaling pathway (66, 67). Activation of sirtuins and reduction of oxidative stress through the use of resveratrol are thought to prevent chronic inflammation (68), but paradoxically, the findings of oral resveratrol for improvement of glucose homeostasis and cardiovascular characteristics are divergent (69), but the feces of mice transplanted and fed with resveratrol were better than those of oral administration (70), which may be related to the route of administration, which reinforces the role of enterobacteria in the amelioration of inflammation-associated Mets. Recent studies have shown that seaweed cellulose (71), seaweed polysaccharides (72), acetate (73, 74), fermented sea buckthorn juice (75), and dry-cured ham (76) inhibit oxidative stress and therefore have great potential in controlling Mets as providing new perspectives in the prevention and treatment of Mets.

### 5.2 Mitochondrial dysfunction

Mitochondria are the main site of biological oxidation and the center of energy metabolism in the organism. At the same time, mitochondria are also highly dynamic organelles that are remodeled through biosynthesis, division and fusion, autophagy, and other processes to achieve mitochondrial homeostasis (77). Mitochondrial dysfunction occurs when mtDNA mutations, kinetic imbalances, and oxidative stress occur in mitochondria. Mitochondrial dysfunction and subsequent excess ROS production promotes insulin resistance through activation of the c-Jun amino-terminal kinase (JNK) and NLRP3 inflammatory vesicles, leading to the inactivation of the insulin receptor substrate (IRS)1/PI3K/serine/threonine kinase (Akt) pathway, which promotes insulin resistance progression (78). The main pathogenic mechanism of Mets is closely related to insulin resistance, and the study of mitochondrial dysfunction is undoubtedly an important target for attacking Mets.

It has been shown that SCFAs such as propionate and butyrate enhance mitochondrial biogenesis (79). SCFA supplementation may prevent colonic inflammation and dysregulated mitochondrial energy metabolism by improving the balance between Treg cells and Th17 cells, increasing mitochondrial ETC activity and oxidative phosphorylation (80), but there are very few studies on SCFA and mitochondrial autophagy, among others. It has been well documented that mitochondrial dysfunction is associated with Mets and that abnormal mitochondrial autophagy leads to impaired mitochondrial function and is involved in the development and progression of Mets (81, 82). It has been demonstrated by flow cytometry analysis that propionate induced Δψ loss, leading to aberrant mitochondrial autophagy (83), and that butyrate restored PRKN expression by blocking RELA nuclear translocation and directly inhibiting HDAC8 in the nucleus, which, in turn, ameliorated mitochondrial autophagy in high-glucose-inhibited neuronal cells (84). Mitochondrial autophagy has many cellular pathways in Mets, such as the classical PINK1/Parkin pathway, the mitochondrial autophagy receptor FUNDC1, and the BINP3/NIX pathway, which are closely associated with Mets-associated metabolic disorders, such as type 2 diabetes (85), obesity (86), heart disease (87), and NAFLD (88), respectively. However, two important proteins related to the regulation of mitochondrial autophagy in glucolipid metabolism are currently underappreciated in the context of Mets. FK506-binding protein 8 (FKBP8), a mitochondrial autophagy-recognizing protein, mediates mitochondrial autophagy and fragmentation (89), which dynamically regulates pancreatic islet β-cells and glucose-stimulated insulin secretion (90). Theoretically, FKBP8 is important in the development of insulin resistance or T2DM. However, there are fewer studies on FKBP8. Similarly, BCL-2-like protein 13 (BCL2L13) is a mitochondrial outer membrane protein involved in mitochondrial division in mammalian cells; BCL2L13 is important in the control of apoptosis, mitochondrial fracture, and the promotion of mitochondrial autophagy (91); BCL2L13 can play a role in the promotion of adipogenesis by regulating mitochondrial autophagy process to maintain mitochondrial quality control (92, 93). BCL2L13 may be a promising biomarker as a potential drug therapeutic target for Mets. However, no relevant studies have emerged with Mets.

### 5.3 Epigenetic changes

Since the common aggregation of obesity cannot be explained by common environmental conditions alone, the heritability of obesity is widely believed to be between 40% and 70%. However, the risk loci associated with BMI revealed in genome-wide association studies can only explain approximately 16% of heritability, even after taking into account their superimposed effects (94). As a result, epigenetic mechanisms have become the focus of obesity research in the past few years to explain inherited deletions. Epigenetic mechanisms describe processes that affect the DNA surrounding the transcription of a gene without changes in the DNA sequence itself. Examples include adding methyl groups to cytosine and then adding guanosine (CpG site) to the sequence. Other mechanisms alter the structure of chromatin, for example, through modifications such as methylation, acetylation, or phosphorylation of histones. Several studies have observed differences in methylation levels at different CpG sites between obese and lean individuals and after weight loss interventions (95–97).

SCFAs achieve epigenetic regulation through HDAC inhibition, increase mitochondrial β-oxidation, and prevent high-fat diet-induced insulin resistance, thereby improving glucose sensitivity and obesity. Epigenetic mechanisms have also been shown to play an important role in the regulation of genes involved in inflammatory processes and have been closely linked to SCFA-producing bacteria and metabolic diseases. SCFAs are thought to influence inflammation and chronic diseases (98), and butyric acid can regulate gene expression by inhibiting HDACs, which, in turn, prevents HDACs from suppressing the expansion of anti-inflammatory Treg cells. Butyrate and, to a lesser extent, propionate induced the differentiation of colonic Treg cells to suppress inflammatory and allergic responses in the gut (44). GPR43 and GPR41 also triggered the inhibition of HDAC 1 (99), which enhanced the apoptosis of activated T cells, thus possessing anti-inflammatory potential. It has been shown that mice lacking Toll-like receptor 4 (TLR4) or under antibiotic treatment display reduced LPS and are protected from systemic lipid infusion and alleviate Mets (100). Surprisingly the inhibition of TLR4 gene transcription can be mediated by DNA methylation and histone deacetylation (101), which are both associated with epigenetic mechanisms and have not been linked to SCFA by any investigator, which is a good therapeutic target.

## 6 Effects of SCFA in components of the inflammatory Mets and its complications

**Figure 4:**
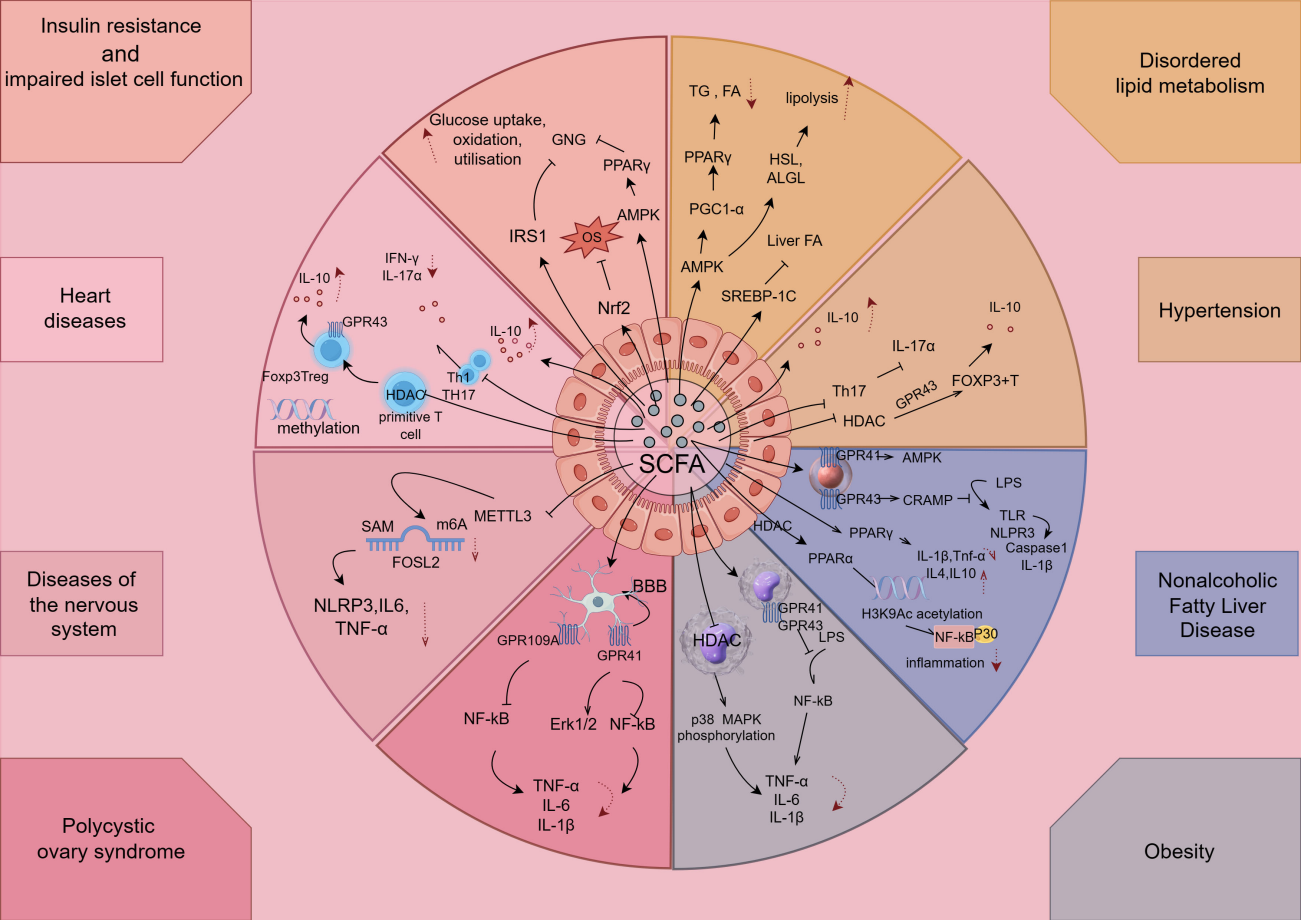
SCFAs exert anti-inflammatory effects in components of Mets. The figure is drawn with Figdraw.com. LPS, lipopolysaccharides; OVA, ovalbumin; TG, triglycerides; NK, natural killer; TH, T-helper cell; Treg, T-regulatory cell.

After SCFA is absorbed by the intestine, part of it is utilized locally as fuel for intestinal epithelial cells, and the other part enters the portal vein to enter the blood circulation, which improves Mets-associated disorders of glucose and lipid metabolism as well as cardiovascular, reproductive, and neurological complications by exerting anti-inflammatory effects (e.g., **Table 1**, **Figure 4**).

**Table 1:** Summary of substances whose target SCFAs improve Mets by inhibiting inflammation.

Veterinary drug	Target point	Findings related to Mets	Reference	Research design
Seaweed cellulose	Antioxidant	Reduces malondialdehyde (MDA) and superoxide dismutase (SOD), enhances total antioxidant capacity (TAC), and inhibits oxidative stress.	(72, 276)	SC was administered to high-fat sugar diet (HFSD)-induced C57BL/6 mice by intragastric gavage at 250 or 500 mg/kg bw/day for 6 weeks.
Acetate circumvents	Inhibition of PDK4/NLRP3 inflammasome	Improves insulin resistance, decreases testosterone and leptin, and increases adiponectin levels; decreases lipid deposition, malondialdehyde, inflammatory mediators (nuclear factor-κB and tumor necrosis factor-α), lactate dehydrogenase, lactate/pyruvate ratio, HDAC, and PDK 4 in skeletal muscle; and increases glycogen synthesis, glutathione, and NrF2.	(73)	Eight-week-old female Wistar rats were divided into three groups (*n* = 6) and treated with drug, letrozole (1 mg/kg) and letrozole plus acetate (200 mg/kg). The drugs were administered orally for 21 days.
Acetate	NF-κB/NLRP3 immune response	Attenuation of hyperandrogenism, apoptosis, oxidative stress and NF-κB/NLRP3 immunoreactivity eliminates renal dysfunction in animals with experimental polycystic ovary syndrome.	(74)	Eight-week-old female Wistar rats were randomized into four groups (*n* = 6) to receive drug, sodium acetate (200 mg/kg), letrozole (1 mg/kg), and letrozole plus sodium acetate. The drug was administered orally once daily for 21 days.
Sea buckthorn juice via fermentation	Flavonoids	Flavonoids promoted interactions between FHJ and probiotics such as *Akkermansia* and Lachnospiraceae. Additionally, FHJ increased SCFAs associated with improvements in multiple sclerosis.	(277)	High-fat dietary chow HFD and BHJ supplements were fed to C57BL/6 mice for 16 weeks.
Xuanwei Ham Proteins	Antioxidant, AMPK/Nrf2 activation	Maintains low serum levels of TC and LDL cholesterol and has high antioxidant activity. This regulation of lipid metabolism and oxidative stress may be related to AMPK/Nrf2 signaling.	(76)	C57BL/6 mice were fed a diet containing casein, raw ham protein (XWH0), or Xuanwei ham protein (XWH1, XWH2, or XWH3) after maturation for 1, 2, or 3 years for 4 weeks.
Propionate and butyrate	Mitochondrial biogenesis	Enhances mitochondrial biogenesis and promotes early neurogenic differentiation of neural stem cells through ROS and extracellular signal-regulated kinase 1/2-dependent mechanisms.	(79)	C57BL/6N male non-littermate mice were fed a high-fat, choline-deficient diet for 14 and 24 weeks.
Butyrate	M6A methyltransferase METTL3, NLRP3 protein	Inhibition of the expression of the m6A methyltransferase METTL3 resulted in a decrease in FOSL2 m6A methylation levels and mRNA expression. In addition, NLRP3 protein expression and inflammatory cytokine (IL-6 and TNF-α) expression were downregulated in KGN cells.	(278)	The human ovarian granulosa cells were treated with several doses of butyric acid (BA) (1.1 mg/mL and 11 mg/mL) in DMEM/F12 media for 24 h in six-well cell plates.
Cedryl acetate ameliorates adiposity	Adipose tissue	Regulation of metabolism-related gene expression in mouse liver (including Pepck, G6Pase, and Fbp1) and epididymal white adipose tissue (including PPARγ, C/EBPα, FABP4, FAS, Cytc, PGC-1α, PRDM16, Cidea, and COX4).	(279)	Three groups of 10-week-old C57BL/6J mice were fed chow, a high-fat diet, or a high-fat diet supplemented with CA (100 mg/kg) for 19 weeks.
Tea catechins	PPARα	Significantly alleviates obesity and low-grade inflammation. Reduces hepatic steatosis and upregulates hepatic peroxisome proliferator-activated receptor alpha (PPARα) mRNA and protein expression.	(280)	Green, oolong, and black tea catechins in high-fat diet (HFD)-fed C57BL/6J mice.
Dietary betaine	miR-378a/YY1 regulating axis	Increasing two major members of SCFAs, including acetate and butyrate, regulates DNA methylation levels in the host miR-378a promoter, thereby preventing obesity and glucose intolerance.	(281)	8-week-old Kunming mice were initially supplemented with a 200-μL combination of four nonabsorbable antibiotics, namely, ampicillin, neomycin, metronidazole, and vancomycin (Sangon Biotech, China) via oral gavage daily (10 mg of each antibiotic per mice per day). After 10 days, the oral gavage was changed to *ad libitum* administration in drinking water (1 g/L ampicillin, 1 g/L neomycin, 1 g/L metronidazole, and 500 mg/L vancomycin) for indicated durations.
Bletilla striata oligosaccharides	Inhibition of Mcp-1 and Cd11c (a macrophage membrane molecule) overexpression in white adipose tissue	Prevents weight gain, reverses glucose intolerance and insulin resistance, and inhibits adipocyte hypertrophy. BO-treated mice also inhibited chronic inflammation and protected the intestinal barrier from disruption.	(282)	Treatment of high-density lipoprotein cholesterol (HFD)-fed 6-week-old C57BL/6 J male mice with BO for 5 months.
SCFA mixtures (acetate, propionate, and butyrate)	Phospho-NF-κB (P-NF-κB) and iba1, and cellular mortality	Significant downregulation of inflammatory cytokines TNF-α, MCP-1, IL-6, and IFN-Υ, reduction of inflammatory mediators phospho-NF-κB (P-NF-κB) and iba1, and cellular mortality in mice infected with Japanese encephalitis virus.	(283)	BALB/c mice on day 10 of life were given intraperitoneal injections of SCFA mixture (acetate, propionate, and butyrate) or PBS for 7 days, followed by JEV infection.
Acetate	Regulation of mitomycin-2 (MFn2)	Decreased inflammation in the ovary (tumor growth factor and nuclear factor-kB), caspase-6, elevated hypoxia-inducible factor-1α, and decreased histone deacetylase-2 (HDAC2). Ameliorates mitochondrial abnormalities observed in rats with polycystic ovary syndrome, elevates adenosine triphosphate synthase and MFn2. Ameliorates ovarian mitochondrial abnormalities.	(223)	Eight-week-old female Wistar rats were randomized into four groups (*n* = 5). Polycystic ovary syndrome was induced with 1 mg/kg letrozole (orally) for 21 days. Afterwards, the rats were administered acetate (200 mg/kg; orally) for 6 consecutive weeks.

### 6.1 Type 2 diabetes

Type 2 diabetes is strongly associated with insulin resistance. The potential mechanisms of intestinal microbial translocation to the pancreas are unknown, and one possibility that has been suggested is via the pancreatic ducts (102), while another may be via the portal vein via the bloodstream from the distal gastrointestinal tract (103). Translocation of flora triggers innate and adaptive immune responses and induces inflammation in colonized tissues or organs; interestingly, translocation of *Enterococcus* and *Escherichia coli* leads to acute pancreatitis (104). Inflammatory diseases, such as inflammatory bowel disease (IBD), are known to be associated with higher intestinal permeability (105), and increased intestinal permeability can also lead to increased commensal bacterial translocation (106). There is a large body of research showing that patients with IBD have a higher risk of developing a number of pancreatic diseases, including type 2 diabetes (107), pancreatitis (108), and pancreatic cancer (109). In terms of metabolic diseases, a national cohort study from Denmark showed that patients with IBD are at a higher risk of developing type 2 diabetes (110), suggesting that intestinal inflammation is strongly associated with insulin resistance. Notably, it was found that mutations in autoimmune-related genes overlap between IBD and type 1 diabetes (T1D), such as protein tyrosine phosphatase non-receptor type 2 (PTPN2) and PTPN22, which negatively regulate T-cell activation (111) and alter the composition of the gut microbiota in IBD patients (112). This suggests that the association between T1D and IBD may involve immune dysfunction and increases the likelihood of overlapping genes between IBD and diabetes.

SCFAs improve insulin sensitivity through multiple pathways. In terms of inflammation, SCFA can inhibit intestinal and systemic inflammatory responses and reduce the release of pro-inflammatory factors, thereby improving the mechanism of insulin action (113, 114). For example, SCFA can enhance the barrier function of intestinal epithelial cells, reduce intestinal permeability, and prevent endotoxin from entering the circulation (115). In addition, acetate, propionate, and butyrate also appear to regulate hepatic glucose metabolism through activation of AMP-activated protein kinase (AMPK) and promotion of PPARγ on gluconeogenesis (116).

Although clinical studies have demonstrated a relationship between SCFA and insulin resistance, these results of clinical interventions have been variable. Several systematic evaluations and meta-analyses suggest that increased intake of SCFA may be beneficial in reducing fasting insulin levels and improving insulin sensitivity (117, 118). However, many trials suffered from poorly considered experimental designs, and future studies need to pay attention to assessing several factors, such as the different types of SCFA (acetic acid, propionic acid, butyric acid, or mixtures thereof), the form of SCFA administration (fiber-rich diets or capsules containing sodium salts or inulin esters), the duration of the intervention, the route of administration (oral or intravenous infusion), and patient adherence.

SCFAs can also play a role in islet cytoprotection, and butyrate treatment of non-obese diabetic mice reduced the proportion of inflammatory/regulatory macrophages through the SHIP-1/PI3K pathway, which suppressed the frequency of diabetogenic IFN-γ+CD8+ T cells in the pancreas and reduced diabetes incidence (119). The addition of SCFAs (especially butyrate) to islet cell culture dishes increased the production of the antimicrobial peptide CRAMP by islet β-cells (119), but the exact mechanism involved in the regulation of the antimicrobial peptide CRAMP through which mechanism, GPCRs or HDAC, is not known and needs to be further investigated.

### 6.2 Disordered lipid metabolism

Aseptic inflammation promoted by a high-calorie or high-fat diet can disrupt the gut microbiota and lead to metabolic endotoxemia, which can trigger activation of innate immunity (120). A growing body of evidence indicates that a specific group of bacterial species are associated with obesity and related metabolic defects such as insulin sensitivity by regulating lipid metabolism and systemic inflammatory responses (121–123). Whereas SCFAs play an important role in lipid metabolism, SCFAs not only participate in lipid metabolism as substrates, but also act as regulators to modulate lipid metabolism. As a member of the fatty acid family, SCFAs provide substrates for lipid synthesis. SCFAs can be converted to acetyl coenzyme A to generate energy through the tricarboxylic acid cycle (124). In addition, acetate, as a precursor for the synthesis of palmitic and stearic acids, in turn promotes hepatic fatty acid metabolism (125). At the same time, acetyl coenzyme A converted by SCFAs can also generate palmitic acid through the action of the cytoplasmic enzyme system, and palmitic acid can be transferred to the mitochondria to promote the formation of triglycerides and be stored in adipose tissue (126). Li showed that butyric acid increased fatty acid oxidation in brown adipose tissue and improved diet-induced obesity and insulin resistance (127). Butyric acid also promotes white tissue browning, morphologically reduces adipocyte size, and increases the number of multicellular adipocytes (126).

In specific inflammation-related mechanisms, the AMPK signaling pathway is involved in lipid metabolism and increases PGC-1α expression in adipose tissue and skeletal muscle (128), and chronic AMPK activation via loss of FLCN induces functional beige adipose tissue through PGC-1α/ERRα, as PGC-1α regulates the transcriptional activity of various transcription factors, including PPARα and PPARγ (129). Furthermore, it has been shown that activation of the AMPK signaling pathway promotes the expression of hormone-sensitive lipase (HSL) and adipose triglyceride lipase (ATGL) as the main enzymes of lipolysis, and promotes lipolysis (130, 131).However, there are findings that are controversial with them, and one study found that SCFAs can reduce protein kinase A (PKA) activity by inhibiting adenylate cyclase (132), which, in turn, leads to dephosphorylation and inactivation of HSL in adipose tissue (133). Similarly, Jocken et al. found that acetate exerts anti-lipolytic effects by inhibiting the phosphorylation activity of HSL in human pluripotent adipose tissue-derived stem cells (134). Surprisingly, however, it was recently found that activation of AMPK in murine hepatocellular carcinoma cells inhibits hepatic fatty acid synthesis via suppression of sterol regulatory element-binding protein 1C (SREBP-1C), a major regulator of hepatic adipogenic gene expression (135), which is a potential therapeutic target.

### 6.3 Hypertension

Hypertension is one of the important components of Mets, and hypertensive patients have higher levels of IL-6, IL-8, and TNF-α in the blood (136). The systemic inflammatory state adversely affects the body’s RAAS system and the vascular endothelial system, leading to elevated blood pressure, which increases intestinal inflammation and permeability in patients (137); it can affect intestinal physiology and decrease the abundance and diversity of intestinal flora, thus decreasing the concentration of SCFAs. SCFAs exert their regulatory functions mainly through the inhibition of HDACs and the activation of Gpr43 and Gpr109a, and intervene in the key factors of kidney and brain through the mechanism of renal–intestinal axis and brain–intestinal axis, which not only control inflammation to regulate blood pressure, but also influence metabolism and immunity to regulate blood pressure. Specifically, in terms of immuno-inflammation, SCFA can achieve the occurrence and development of hypertension by regulating effector T cells, helper T cells (Th), and regulatory T cells (Treg).

In terms of effector T cells, in the active immune response state, SCFAs promote effector T-cell generation and, at the same time, can increase the cytotoxicity of CD8+ T cells and the ability to generate IL-17, whereas in the physiological state, SCFA promotes the body’s immune tolerance to effector T cells through the increase in the generation of IL-10, which, in turn, can have an inhibitory effect on chronic inflammatory responses in hypertension (138). In terms of Th, infusion of Ang II into the spleens of wild-type mice increased the level of CD4+ effector memory T cells (CD44+CD62−) and decreased the level of CD4+ initial T cells (CD44−CD62+), resulting in the release of the inflammatory factors IL-17a and IFN-γ from Th17 and Th1, respectively. This, in turn, promotes the development of hypertension and its target organ damage, which is reversed by propionate supplementation (139). This suggests that propionate can ameliorate the inflammatory response by inhibiting the increase in the number of effector T cells and Th17 cells, thereby decreasing IL-17a secretion. With regard to Treg, SCFA increases histone acetylation by inhibiting HDAC, which can differentiate primitive T cells into Treg and increase Foxp3 expression via Gpr43, thereby increasing Fox P3+ T-cell activity and IL-10 production (140), producing an anti-inflammatory effect and thus alleviating the progression of hypertension (139). It has also been suggested that its antihypertensive properties may be due to the binding of SCFA to Gpr43 in the lamina propria of intestinal epithelial cells, resulting in the polarization of T cells and their conversion to Treg, which then migrate and accumulate in the renal cortex (141). In addition, the proportion of methylated regions in genes related to Treg function increased to different degrees, and supplementation with acetate altered the level of DNA methylation in Treg-activated regions, suggesting that SCFA regulates the methylation of functional genes in T cells and thus activates Treg. However, Gill (142) found that short-term increases in systemic acetate and propionate levels did not alter Treg levels in mice, suggesting that the concentration of SCFA has a strong influence on the outcome of their effects in immunization. However, different types of SCFA have different effects on blood pressure, and propionate may have a better effect on blood pressure control compared with butyrate. In spontaneously hypertensive rats, butyrate treatment did not affect intraocular pressure and caused only a transient decrease in blood pressure (143). In contrast, propionate significantly reduced systolic and diastolic blood pressure in angiotensin (Ang) II mice, ameliorating systemic inflammation, cardiac damage, and vascular dysfunction (144).

### 6.4 Obesity

Obesity-induced systemic inflammation can contribute to the development of a number of non-communicable chronic diseases, such as type 2 diabetes, chronic liver disease, arthritis, and certain types of cancer (145). In obesity, two main mechanisms are involved in systemic inflammation. First, inflammation is driven by adipose tissue macrophages (ATMs). Significant enlargement of adipose tissue due to excess energy intake stimulates macrophage polarization from the anti-inflammatory M2 type to the pro-inflammatory M1 type (146). M1-type macrophages cause further inflammation by producing pro-inflammatory cytokines such as TNF-α and IL-6 (147). In addition, the inability of M1-type macrophages to buffer lipids leads to lipid spillover into the circulation and activation of pro-inflammatory cascades (147). Second, systemic inflammation in obesity can also be caused by metabolic endotoxemia, a condition characterized by elevated levels of circulating endotoxins, including LPS. In obesity, increased intestinal permeability due to dysbiosis of the intestinal flora affects the tight junction proteins in the intestinal lumen, which, in turn, leads to infiltration of LPS into the blood circulation (148). Elevated metabolic danger signals, free fatty acids (FFAs), and the bacterial danger signal, LPS, activate the NF-κB and MAPK pro-inflammatory pathways, which are regulators of systemic inflammation in obesity, by binding to TLRs (149), resulting in the transcription of pro-inflammatory cytokines, such as TNF-α, IL-6, and IL-1β, which initiate systemic inflammation (150).

To date, one of the most effective treatments for obese chronic low-grade systemic inflammation is weight loss (151). Bariatric surgery results in significant weight loss, leading to a significant reduction in the systemic inflammatory response (152), with significant reductions in the pro-inflammatory cytokines CRP, TNF-α, and IL-6 (153). SCFAs are a non-invasive, novel, alternative treatment for systemic inflammation in obesity compared to the invasive treatment of bariatric surgery. Whereas SCFAs can inhibit LPS-induced inflammation (154), in addition to that, SCFAs have many anti-inflammatory mechanisms (155, 156). Treating SCFA modulates the expression of FFAR and HDAC genes involved in key inflammatory pathways in monocytes and ATMs of obese subjects. First, SCFAs play an important role in obesity systemic inflammation by regulating FFARs. Their key targets are free fatty acid receptor (FFAR) 2, which is mainly expressed in immune cells, and FFAR3, which is mainly expressed in pancreas, spleen, and adipose tissue, both of which are associated with metabolic diseases such as obesity and T2DM (156). FFAR2 and FFAR3 are upregulated by LPS stimulation in monocytes and macrophages (157). Second, SCFAs can inhibit HDACs, leading to downregulation of NF-κB and MAPK pro-inflammatory pathways (156). Jeong found that inhibition of HDAC1–3 reduced LPS-induced phosphorylation of p38MAPK, which, in turn, reduced LPS-induced expression of TNF-α and IL-1 β (158). Propionate- and butyrate-mediated inhibition of HDACs in SCFAs plays an important role in the downregulation of the MAPK pro-inflammatory pathway. The SCFAs butyrate and propionate interact predominantly with HDACs 1 and 2, which are located in the nucleus, in addition to HDACs 3, 4, 5, 6, 7, and 9, which are shuttled between the cytoplasm and the nucleus (159). Butyric and propionic acids, considered to be the most potent HDAC inhibitors, have previously been shown to inhibit TNF-α production and NF-κB activity, whereas evidence suggests that acetic acid has little ability to inhibit HDAC activity (45, 160, 161).

The type and concentration of SCFA also affect the concentration of inflammatory factors in obese patients, and several studies have shown that butyrate, propionate, and acetate all significantly reduced TNF-α and IL-6 production in LPS-stimulated monocytes from obese subjects. Only the degree of inflammatory factors reduced by different SCFA species varied, with acetate and butyrate significantly reducing TNF-α production by LPS-stimulated ATMs, and propionate and butyrate significantly reducing IL-6 (162, 163). However, Cox showed that SCFAs (0.2–20 mmol/L) were effective in reducing LPS-induced TNF-α in PBMCs, but not in human monocytes. Few studies have measured the effect of SCFAs on obese ATMs. Al-Lahham showed that 3 mM propionic acid significantly reduced LPS-stimulated TNF-α responses in ATMs (164). However, some studies did not observe an inhibitory effect of propionate on TNF-α production, which may be related to the concentration of propionate (163). Since ATMs are less responsive to LPS stimulation than monocytes and adipose tissue is difficult to obtain, there are few studies related to the effect of SCFAs on inflammation in human ATMs, but this study is related to the important mechanism of chronic inflammation in obesity, so attention should be paid to the techniques of adipose tissue extraction and the effect of SCFAs on inflammation in ATMs.

### 6.5 Nonalcoholic fatty liver disease

The liver is involved in fat synthesis and is therefore closely related to Mets. The intestine and liver are closely connected through the portal vein and the biliary system. Enteric substances such as digested food fragments (amino acids, lipid fragments, and monosaccharides), intestinal microbial products, and exogenous toxins enter the liver via the portal vein (165). Animal studies have also demonstrated that during high-fat diet-induced NAFLD, there is a gradual increase in LPS levels and changes in the composition of the intestinal flora (166) and that the exacerbation of NAFLD to NASH is also associated with an increase in LPS levels (166). It can be seen that rapidly proliferating bacteria and their metabolites enter the portal vein through the damaged intestinal wall, and the inflammatory response caused by the level of LPS, which activates the cascade immune response and causes hepatic inflammation, is the key to the occurrence as well as further deterioration of NAFLD. Intestinal SCFAs can reach the liver through the hepatic portal vein. *In vivo* and *in vitro* studies have found that the anti-inflammatory effects of butyrate on the liver are mainly mediated through Kupffer cells. *In vivo* in rats, butyric acid infusion through the portal vein enhances the production of the immunosuppressive arachidonic acid metabolite, prostaglandin E2 (PGE2), by Kupffer cells. *In vitro*, butyric acid supplementation of Kupffer cells also increased PGE2 production and inhibited TNF secretion (167).

Two inflammatory mechanisms can be found in NAFLD mouse models, both of which can be mediated by microbial components such as LPS, and they can be transferred from the intestinal lumen to the liver through the portal vein. One mechanism is that they are recognized by pattern recognition receptors such as TLRs in the liver, e.g., TLR4, leading to hepatic inflammation, hepatocellular injury, and liver fibrosis (168). Similar pathways have been suggested to contribute to the development of NAFLD. Activation of inflammatory vesicles, especially the NLRP3 inflammatory vesicle, is thought to be another trigger of the hepatic inflammatory response to LPS. Activation of NLRP3 in the liver leads to activation of caspase 1 and production of IL-1β and several other inflammatory cytokines, ultimately leading to programmed cell death, inflammation, and fibrosis. NLRP3 was significantly upregulated in the livers of NASH patients compared with steatosis alone (169), and hepatic inflammatory vesicle fractions correlated with ALD activity (170). On the other hand, NLRP6 and NLRP3 inflammatory vesicle defects regulate the gut microbiota, leading to gut barrier dysfunction and exacerbating steatohepatitis in mice (171).

SCFAs may exert an anti-inflammatory response through activation of GPR41/43 and AMPK and inhibition of HDACs, which is beneficial for the treatment of NAFLD (172). In rodents, enteral administration of acetate and butyrate has been shown to induce the expression of beta-defensin and histone inhibitor-related antimicrobial peptides in intestinal epithelial cells in a GPR43-mediated manner as a means of protecting the intestinal barrier and organizing the inflammatory response induced by, among others, LPS (173). Activation of GPR43 on adipocytes inhibits lipolysis and reduces plasma free fatty acid levels in the binding mechanism of SCFAs to the GPRs (174). Similarly, GRP41/43 knockout mice exhibit a smaller trend toward obesity (175, 176).

In addition to the immuno-inflammatory mechanisms associated with binding to GPRs, SCFAs enter the liver directly through the portal vein and promote triglyceride synthesis (from acetic acid) and gluconeogenesis (from propionic acid), which have been implicated in the development of NAFLD (177). Rau et al. (178) found that patients with NAFLD had a significantly higher abundance of SCFA-producing bacteria in their feces and that higher levels of fecal SCFAs (acetate and propionate) were associated with higher Th17/Treg cell ratios in the peripheral blood, convincingly suggesting that the gut microbiome may modulate the immune response and contribute to disease progression through the production of greater amounts of SCFAs. Compared to healthy human controls, patients with NAFLD and NASH (179) had increased concentrations of SCFAs in their feces, as well as an increased abundance of bacterial flora involved in their production, and the immune cell profiles of both were altered. In detail, this increased fecal SCFA observed in NASH was associated with a decreased number of resting Treg cells (CD4+CD45RA+CD25+) and a higher ratio of Th17 cells to resting Treg cells in the peripheral blood, which is a systemic immune feature observed in NASH (180).

SCFA also prevents and ameliorates NAFLD by protecting hepatocytes, and studies suggest that butyric acid has two mechanisms of action for PPAR receptors to protect hepatocytes. On the one hand, butyric acid prevents the decreased expression of PPAR-γ in the liver, which promotes fatty acid uptake, increases insulin sensitivity (181), and decreases the expression of pro-inflammatory cytokine gene (IL-1β and TNF-α) macrophage infiltration-specific markers F4/80, which leads to a significant reduction of F4/80+ cell infiltration and anti-inflammatory cytokines in the liver of mice (IL-4 and IL-10) and activation of hepatic Kupffer cells, thereby preventing liver injury and inflammation in NAFLD mice (182). On the other hand, butyric acid acts as an HDAC inhibitor, upregulates PPAR-α expression, and promotes the binding of the NF-κB classical subunit p65 by increasing the acetylation of H3K9Ac on the PPAR-α promoter, thereby inhibiting the inflammatory response (183). In addition, the finding that SCFAs can reduce the expression of hepatic genes involved in NAFLD, mainly lipogenic genes such as those encoding acetyl coenzyme A carboxylase, fatty acid synthetase, and sterol regulatory element-binding protein 1c (cholesterol regulatory element-binding protein-1c (184)) through the action of HDAC inhibitors, provides important mechanistic insights. The concern, however, is that butyric acid is strongly affected by antibiotics, and it has been shown that butyric acid levels are significantly reduced in the liver of mice treated with antibiotics early in life, leading to impaired IL-18 signaling, which, in turn, inhibits mitochondrial function and maturation of liver-resident natural killer (NK) cells, whereas ingesting dietary butyric acid can, in a GPR109A-dependent manner, stimulate IL-18 production in Kupffer cells and hepatocytes to reverse this process (185). However, the mechanisms involved are unclear and need to be continued to be explored by researchers.

### 6.6 Heart diseases

Atherosclerosis (AS) is a complex disease with multiple etiologies, and one of the factors contributing to AS is metabolic disorders. AS is an inflammatory disease that is associated with chronic vascular inflammation, and its most common pathologic process leads to cardiovascular disease. The earliest event in AS is increased adhesion of monocytes to endothelial cells, which is mainly regulated by vascular inflammatory factors, including cytokines such as IL-6, chemokines such as IL-8 and monocyte chemotactic protein-1, as well as endothelial adhesion molecules such as vascular cell adhesion molecule-1 and intracellular adhesion molecule-1. In clinical studies, less endothelial activation and less low-grade inflammation have been associated with high fiber depletion, possibly due to the production of SCFA. SCFAs have recently emerged as important signaling molecules that regulate a variety of responses in the cardiovascular system (186), and SCFAs have been shown to play a beneficial role in decreasing endothelial activation, resulting in decreased cytokine production and adhesion molecule expression (187). It may regulate endothelial function by inhibiting HDAC and/or activating GPRs (160).

SCFAs also play an important role in immune system regulation in modulating cardiovascular disease. Notably, it has been shown that both HDAC and NF-κB contribute to immune and inflammatory responses (188), whereas butyrate inhibits the activation of HDAC and NF-κB in macrophages (189). SCFAs are also involved in anti-inflammatory responses by upregulating anti-inflammatory cytokines and downregulating pro-inflammatory cytokines. For example, binding of SCFAs to FFAR2 and GPR109A in intestinal epithelial cells stimulates K+ efflux and hyperpolarization, leading to activation of the inflammatory vesicle-activating protein NLRP3, which, in turn, induces the release of IL-18, contributing to the maintenance of integrity, repair, and intestinal homeostasis (190). Butyrate increases protein acetylation and TGF-β production in the intestinal epithelial cells, leading to a decrease in IL-8 production (191) and an increase in anti-inflammatory Treg cells in the intestinal epithelial cells, respectively (192). In human mature dendritic cells, butyric and propionic acids appeared to reduce the release of pro-inflammatory chemokines such as CXCL11, CXCL10, CXCL9, CCL5, CCL4, and CCL3, as did inhibiting the expression of LPS-induced cytokines, including IL-6 and IL12p40. In addition to the modulation of cytokine production, SCFAs inhibited, by lowering the luminal pH, the growth of pathogenic bacteria (193). Finally, SCFAs, especially butyrate, can contribute to host defense by inducing the antimicrobial protein histone inhibitor IL-37 (194) and increasing the levels of T regulatory cells in the gut (44). In summary, we can infer that SCFAs can exert benefits in metabolic cardiovascular disease characterized by dysregulation of blood pressure, glucolipid metabolism, inflammatory response, and/or gut barrier integrity. Indeed, several studies have demonstrated the benefits exerted by SCFAs in metabolic cardiovascular disease.

It is worth mentioning that recent studies on SCFA for polycystic ovary syndrome (PCOS) in relation to inflammation in the heart have become progressively more frequent, and it has been found that acetate can reverse cardiac energy depletion, alleviate nitric oxide (NO/eNOS) deficiency, elevate SIRT-1/HIF-1α levels, and decrease CTGF/TGFβ-1 in an experimental PCOS model by inhibiting HDAC2, oxidative stress (malondialdehyde)/inflammation (NF-κB/SDF-1) markers, and plasma troponin T levels (195). Acetate has also been found to prevent cardiac inflammation in a rat model of PCOS by inhibiting PCSK9 and NF-κB-dependent mechanisms (196).

### 6.7 Diseases of the nervous system

Mets and major depressive disorder are two of the most serious disorders worldwide, often reported to have a high comorbidity rate, and studies have demonstrated a strong correlation with inflammation (197). In addition to influencing material metabolism, the gut microbiota affects the nervous system by regulating the hypothalamic–pituitary–adrenal axis and producing neuroactive substances (198). The interaction between the central nervous system (CNS) and the gut flora is known as the flora–gut–brain axis. A growing body of research suggests a complex interaction between the microbe–gut–brain axis and psychiatric disorders such as anxiety (199), obsessive–compulsive disorder (200), and depression (201). One study found that blood levels of cytokines, including IL-6 and TNF-α, were significantly higher in depressed patients than in healthy controls (202), and that these pro-inflammatory cytokines, especially IL-6, IL-1β, and TNF-α, promote the production of Th17, which has been implicated in depression and other CNS disorders closely associated with them (203). The products of Th17 cells, IFN-γ, and IL-17A contribute to microglia proliferation, polarization, and activation (204, 205), which could promote neuroinflammation, whereas microglia are the main source of cytokines in all glial cells in the CNS (206). Microglia are polarized to an M1 phenotype, releasing ROS and pro-inflammatory cytokines, including IL-1 β, IL-6, and TNF-α (207).

SCFAs are the main mediators of the microbial–gut–brain axis in the pathophysiologic process of depression. Chronic stress accompanied by dysbiosis of the gut flora interferes with the metabolism of SCFAs and accelerates the dysfunction of the microbial–gut–brain axis in depression. SCFAs have neuroprotective roles and are involved in the complex biological mechanisms and pathological processes involved in the onset and progression of depression including chronic cerebral hypoperfusion, neuroinflammation, epigenetic modifications, and neuroendocrine alterations, which are summarized in this section, with a focus on their inflammatory contributions.

Bilateral common carotid artery occlusion (BCCAO) has been used to establish a rat model of depression, and recent studies have evaluated the role of SCFAs in depression. Xiao et al. demonstrated that the intestinal flora of BCCAO rats was disturbed, and that the reduction of SCFA-producing flora led to cognitive impairment and depression-like behaviors in the hippocampus of BCCAO rats. Xiao et al. showed that the intestinal flora of BCCAO rats was disturbed, and the reduction of some representative SCFA-producing flora led to the reduction of SCFA in the hippocampus of BCCAO rats, which, in turn, led to cognitive deficits and depressive-like behavior (208). In contrast, researchers have found that SCFAs can reduce hippocampal neuroinflammation and neuronal apoptosis induced by depression models through inhibition of NF-κB and activation of the ERK1/2 pathway, with concomitant improvements in cognitive decline and degenerative processes (208).

Gut microbial-derived SCFAs can play a crucial role in anti-inflammatory actions through direct and indirect mechanisms, as they maintain gut–brain permeability and sustain inputs to the CNS to maintain microglia homeostasis (209). In the CNS, propionate protects the blood–brain barrier (BBB) via FFAR3 on the surface of endothelial cells (210), whereas in the intestinal barrier, SCFAs, especially acetate, propionate, and butyrate, are more protective of the intestinal barrier (211). Butyrate enhances the expression of aromatic hydrocarbon receptor and hypoxia-inducible factor 1α (HIF-1α), and upregulates the levels of IL-12 (212), a protective cytokine that helps to resist inflammatory stimuli and maintain intestinal homeostasis, through modulation of mammalian target proteins of rapamycin and signal transducers and activators of transcription. These findings suggest that SCFAs exert an inhibitory neuroinflammatory effect from an indirect mechanism by maintaining intestinal homeostasis. SCFAs have direct anti-inflammatory effects on microglia proliferation (213) and activation by binding to FFARs. SCFAs could bind to GPR41 on microglia, modulate microglia proliferation and inflammation, and inhibit pro-inflammatory signaling pathways by inhibiting NF-κB and activating Erk1/2 (208). In microglia, butyric acid also activates the GPR109A-mediated signaling pathway to downregulate the NF-κB signaling pathway, inhibits the production of pro-inflammatory enzymes (inducible nitric oxide synthase and cyclooxygenase-2) and pro-inflammatory cytokines (TNF-α, IL-1β, and IL-6) in microglia, and prevents the onset and progression of neuroinflammation. Markers reflecting the anti-inflammatory status of microglia IL-10 and CD26 were elevated after butyrate treatment, suggesting a neuroprotective effect of SCFAs *in vivo (* 214, 215). SCFAs also enhance mitochondrial biogenesis (79) by protecting hippocampal neurons from damage to mitochondrial membrane potential and ROS accumulation (216). It follows that complex interactions between neuroinflammation and anti-inflammation via the SCFA-mediated microbiota–gut–brain axis are involved in the pathophysiology of depression.

### 6.8 Polycystic ovary syndrome

Recent studies have shown that chronic inflammation is a risk factor for PCOS, and patients with PCOS are in a state of chronic low-grade inflammation (217), in which inflammatory factors, such as IL-6 and TNF-α, are consistently elevated, and this chronic low-grade inflammation promotes the development of ovarian and metabolic dysfunction in PCOS (218). In addition, higher concentrations of these cytokines and chemokines can lead to the development of reproductive abnormalities with multiple negative effects. On the one hand, abnormal expression of inflammatory factors in the peripheral circulation and ovarian tissues of patients with PCOS induces immune dysfunction and ovulation disorders (219). Dysfunction of the immune system affects follicular development or ovulation (220). On the other hand, inflammatory factors are increased in patients with PCOS, which alters the level of AMH and leads to disorders of glycolipid metabolism (221). Thus, suppression of inflammatory responses is very necessary.

With the deepening research on the pathogenesis of PCOS and its chronic inflammatory state, it has been found that a variety of signaling pathways are involved in the initiation and progression of inflammation (222), among which the more important ones such as PI3K/AKT, MAPK, and AMPK signaling pathways, and the SCFAs have anti-inflammatory roles in PCOS, but these signaling pathways have been reported less frequently in the study of SCFAs. Recent studies have shown that SCFA in PCOS inflammation-related currently has these regulatory mechanisms, such as NF-κB (195, 223), NLRP3 inflammatory vesicles (73, 224), and gamma aminobutyric acid (GABA) (225), but the specific inflammatory pathway mechanisms have not been specifically studied. However, surprisingly, it has been found that SCFAS play an ameliorative role in PCOS to ameliorate inflammation through epigenetic mechanisms. N6-methyladenosine (m6A) is one of the most common forms of RNA modification in mammals (226). Researchers find that m6A modifications can affect cellular inflammation by regulating inflammation-related genes (227). For example, the RNA methyltransferase METTL3 regulates the NF-κB inflammatory pathway by upregulating the level of m6A modification of TRAF6 (228). The addition of butyric acid led to a decrease in FOSL2 m6A methylation level and mRNA expression through inhibition of METTL3 expression and was accompanied by a decrease in NLRP3 and inflammatory factors IL-6 and TNF-α expression, whereas FOSL2, as an AP1 family member, participates in the immune response and promotes the expression of inflammatory factors (229). Furthermore, it has been shown that *Clostridium perfringens* reduces METTL3-mediated m6A modification by inhibiting the Hippo pathway and activating the Yes-Associated Protein (YAP) signaling pathway (230). Butyric acid activates YAP in human intestinal smooth muscle cells (231). However, whether butyric acid inhibits METTL3 through the YAP pathway or affects METTL3 expression through other proteins requires further study.

### 6.9 Stroke

It has been shown that stroke may alter the composition of the gut flora, trigger inflammatory responses and microglia activation, and induce dysbiosis of the gut microbiota, which may influence the deterioration of functional stroke outcomes (232, 233). However, commensal bacterial colonization has a protective effect on the brain after stroke (232), and decreasing the number of pathogenic bacteria that inhibit neuronal apoptosis, oxidative stress, and cerebral infarct volume and increasing the number of beneficial bacteria can prevent neurological deficits (234), in which SCFAs play an important role. SCFAs are mediators between the microbiome and the brain and help to regulate prognostic outcomes after ischemic stroke (235). The gut microbiota plays an important role in microglia function, especially through SCFA as a mediator of microbiota–microglia communication (233).

SCFAs regulate microglia inflammation, glucose metabolism, maturation, and activation through activation of GPCRs or inhibition of HDACs, and affect the maintenance of the BBB (236). Studies have shown that the lack of butyrate-producing bacteria and low fecal butyrate levels are possible factors for increased risk of stroke (237). In a rat model of MCAO, researchers found that the gut microbiome stimulated a protective immune response in the brain after stroke, providing evidence that the gut microbiome plays a protective role in the brain region after experimental stroke (232). SCFAs have been identified as key regulators of intestinal immune cells, and in the context of neuroinflammation, SCFAs alter the balance between pro-inflammatory Th1 and Th17 cells and anti-inflammatory Treg cells (238). In mice, low levels of SCFAs, especially butyrate, were observed during middle cerebral artery occlusion (239), whereas high levels of gut microbiota metabolites, especially butyrate, acetate, and propionate, improved prognosis after ischemic stroke (240).

Several studies have demonstrated the contribution of SCFA to BBB integrity and glial function in ischemic stroke animals. Butyrate improved neurological function and significantly reduced ischemic lesions in aged rats after ischemic stroke. In addition, butyrate reduced the expression of occludin and ZO-1, thereby favoring the reduction of BBB permeability (241). Some scholars investigated the effects of sodium butyrate on ischemic stroke in middle-aged rats and found that treatment with sodium butyrate further inhibited the MCAO-induced increase of IL-1β, IL-17A, and IL-18 in the ischemic hemispheric brain lysates (cortex and striatum), and reduced the ischemia-induced upregulation of IL-1β and IL-18 in the circulation, which suggests that sodium butyrate, as an HDAC inhibitor, has a powerful anti-inflammatory effect and can exert neuroprotective effects (242). In another experimental animal model using adult male rats, high levels of SCFAs, particularly sodium butyrate, reduced infarct volume and improved neurological function and attenuated apoptosis through activation of the PI3K/Akt pathway at 24 and 72 h after MCAO (243). A study in male and female rats demonstrated that microbiota-derived SCFA modulate post-stroke recovery by affecting systemic and brain-resident immune cells, optimizing recovery and cortical reorganization, modulating plasticity and synaptic function, and improving motor function (244). It has been shown that a young microbiome can be restored even a few days after ischemic stroke, largely through the action of SCFA to reduce inflammation and promote recovery in older animals (245). Although there are many studies on the effectiveness of SCFA for stroke treatment, there are many gaps in the specific mechanisms and associated inflammatory-immune aspects of the pathways, and future studies could explore the exact mechanisms by which SCFAs exert their beneficial effects after stroke.

### 6.10 Inflammatory bowel disease

IBD, including Crohn’s disease and ulcerative colitis, is characterized by an abnormal inflammatory response in the components of the intestinal flora that play an important role (246). Disturbed and ecologically dysfunctional intestinal flora is a typical feature of IBD, where butyrate-producing bacteria (e.g., *Faecalibacterium*, *Roseburia hominis*, and *Bifidobacterium*) are in lower abundance, resulting in reduced butyrate levels (247, 248). It has been shown that inflammation reduces the responsiveness of the intestinal epithelium to butyrate and the ability to uptake butyrate in patients with IBD (249). Thus, intestinal inflammation may reduce the levels of butyrate-producing bacteria and the utilization of butyrate. Recent evidence suggests that healthy individuals at high genetic risk for IBD have reduced numbers of *Roseburia* spp. in their gut flora (250). Mouse studies have shown that the anti-inflammatory effects of *F. prausnitzii* in experimental IBD are directly mediated by butyrate downregulation of the pro-inflammatory IL-6–STAT3–IL-17 signaling pathway through HDAC1 inhibition and transcriptional repression that simultaneously promotes Foxp3 expression, thereby maintaining Treg cells (251). Thus, dysregulation of SCFA levels in the gut may be an important susceptibility factor for IBD.

SCFA can suppress intestinal inflammation by inhibiting excessive signaling by TLR. High intake of dietary fiber increases the level of SCFA in the intestine and effectively reduces the degree of TLR-mediated inflammation (252). It is important to note that TLR4 and TLR2 may be key targets of SCFA in preventing IBD (253). In particular, sodium butyrate, an HDAC inhibitor, can inhibit the expression of TLR4 (254). In IBD patients, butyrate has also been found to inhibit TLR2-mediated inflammatory factor release (255). Butyrate also reduces the expression level of junctional proteins (256). In addition, SCFA activates NLRP109 inflammasome by binding to the receptors GPR3 and GPR43A, ultimately maintaining intestinal health in mice (257). Further studies have shown that SCFA maintains intestinal health by regulating NLRP3 inflammasome assembly and attenuation.

Epidemiologic evidence suggests that gut flora disruption due to antibiotic use early in life is associated with an increased risk of developing IBD (258), particularly in the first year of life, as antibiotic use may cause intense and prolonged microbial disruption at a critical time and have long-term effects on the immune system, increasing its risk of future IBD. Further evidence (259) suggests that SCFA production is more affected by antibiotics, with studies in mice showing that SCFA levels are significantly reduced during antibiotic use, and that rebuilding of the flora after antibiotic treatment leads to overactivation of intestinal macrophages, which, in turn, leads to a long-term pro-inflammatory T-cell response. In this model, supplementation with SCFAs, specifically butyrate, prevented macrophage dysfunction and eliminated pro-inflammatory T-cell responses.

SCFA enemas have been shown to be effective in reducing symptoms in a subgroup of patients with ulcerative colitis (260). Butyric acid enemas also reduce the Disease Activity Index in these patients, but subsequent trials have demonstrated minimal effects on colonic inflammatory parameters (261). A preliminary trial investigated the efficacy of encapsulated butyrate as an adjuvant to conventional therapy in maintaining remission in patients with Crohn’s disease and ulcerative colitis (262). Adjunctive butyric acid therapy reduces fecal levels of the intestinal inflammatory marker calreticulin, stimulates the growth of butyric acid-producing bacteria, and improves quality of life in patients with ulcerative colitis. It is important to note, however, that SCFA alone is unlikely to be a universal solution for diseases associated with microbial ecological dysbiosis, and not all nutritional strategies known to be effective in Crohn’s disease have been associated with elevated butyrate or SCFA levels. For example, the first-line treatment for Crohn’s disease in children is pure enteral nutrition, an approach that results in reduced microbial diversity and lower butyrate levels (263). Thus, further research is needed to determine whether strategies that increase SCFA will improve overall treatment outcomes (264).

## 7 Prospects for treatment and challenges

With the current advances in the field of intestinal flora research, modulation of SCFA levels holds promise for use in prevention strategies for inflammatory Mets and maintenance of remission of disease progression. There are many ways to increase SCFA levels in the gut, the most advanced of which are butyric acid administration and modulation of butyric acid metabolism using prebiotics and probiotics. Other routes such as specific diets, fecal bacteria transplantation, and genetically modified bacteria administration have also reached the preclinical or clinical trial stage.

### 7.1 Direct supplementation of SCFA

Oral (262)or rectal administration (265) increases local butyric acid levels or suppositories, and enema application results in increased levels of intestinal luminal and portal venous butyric acid. However, this process does not increase butyric acid levels in the peripheral blood due to a first-pass effect in the liver. Whereas probiotics have received increasing attention in recent years, there have been reviews summarizing information about *F. prausnitzii*, *R. hominis*, and *Clostridium* spp. A large number of studies have been conducted on the role of probiotics in modulating intestinal inflammation, not the least of which are beneficial aspects for Mets (33). Thanks to advanced technologies in macrogenomics and metabolomics, researchers can improve the application of prebiotics and probiotics by analyzing and predicting metabolic networks and other interactions between individual bacterial species (266). Surprisingly, this allows one to go beyond traditional natural strains and obtain genetically engineered probiotics through transgenic technology, such as *E. coli* strains producing increased levels of butyric acid, which have demonstrated efficacy in decreasing disease activity and intestinal damage in colitis models (267). However, the relative effectiveness of genetically modified probiotics compared to natural probiotics is unknown, and there is a lack of clinical trials in this area (268).

### 7.2 Indirect complementary therapies

Fecal flora transplantation aims to replace dysbiotic flora with *healthy* flora. In the context of Mets, transferring the gut flora of a lean individual to a patient with Mets, there have been several studies demonstrating its mechanism of action and clinical efficacy, such as epigenomic effects on host immune cells through methylation of AFAP1, which can lead to improvements in insulin resistance and mitochondrial function. However, compared to diet and prebiotics and probiotics, fecal flora transplantation has little appeal in patients with Mets, and thus dietary interventions offer a more viable pathway. The use of a diet rich in plant fiber may also enhance the growth of SCFA-producing bacteria (269) or be enriched with fermentable prebiotics such as resistant starch (270). In the case of Mets, maternal metabolism is closely related to that of her offspring due to the genetic nature of Mets, and it has been shown that maternal high-fat diet exacerbates inflammatory responses and Mets, disrupts intestinal barrier function, and alters the gut microbiota of the offspring (271). Therefore, intervening in maternal SCFA levels during pregnancy is gradually becoming a research direction for future development, but it is often unethical to test the effects of pharmaceutical compounds or other therapies on pregnant women; thus, the therapeutic benefits of pregnancy-specific diets may be substantial. Several recent studies have demonstrated direct beneficial effects on offspring and alteration of cognitive and social deficits (272) in offspring through a high-fiber diet by promoting Treg cell differentiation (273). In this context, targeting bifidobacteria may be a therapeutic option, as they can utilize human milk oligosaccharides in breast milk to generate SCFAs, thereby preventing systemic inflammation and immune dysregulation (274). It has also been shown that preventing allergies and atopy by supporting bifidobacteria or a high-fiber diet during pregnancy has a strong potential to counteract the intergenerational effects of microbial dysbiosis that may be induced by antibiotics during pregnancy or the early postpartum period (275).

Since SCFAs are involved in both substance metabolism and enterobacterial products, it is unclear whether the benefits of the SCFA-centered therapies described above are related only to the presence of SCFAs or microbial metabolites themselves. In the future, we can focus more on the mechanistic level and use HDAC inhibitors or GPCR ligands more selectively to study these biological effects, to find more fine-grained targets, to find out the specific mechanism of their action with the help of SCFA, and to treat inflammatory Mets by targeting interventions through their biological effects at a higher level.

## 8 Conclusion

Considerable progress has been made in the last decades to better understand the fundamental role of gut flora in health and disease. It is now well established that SCFA, a gut flora derivative, is important in regulating inflammation-associated factors in Mets and its complications such as NAFLD, PCOS, and neurological and cardiovascular diseases. Numerous efforts have been made by basic and clinical translational researchers to determine whether supplementation with SCFA can alleviate or even reverse Mets. In this paper, we summarize the pathways of effect of SCFA in modulating immuno-inflammation in inflammatory Mets; analyze the pathomechanisms of obesity-induced chronic low-grade inflammation leading to oxidative stress, mitochondrial dysfunction, and epigenetic alterations; and itemize the mechanisms of inflammation in the various components and complications of Mets and the roles of SCFA therein; furthermore, we highlight the future directions of research and knowledge gaps.

However, there are still many obstacles and problems from laboratory to clinical applications, especially in terms of the precise effects of different types and concentrations of SCFA in different components and complications, and the sensitivity of different cells to SCFA. Although the effects exerted by the metabolites of specific flora can be analyzed by the latest technology, the substance activity and duration are also issues to be taken into consideration, and the discovery of different targets of SCFA, the use of their targeting mechanisms to guide the treatment of new drugs, and the collaboration of multiple fields including metabolism, microbiology, immunology, genetics, and therapeutic diets will determine the routes of administration and dosages to obtain SCFAs as an anti-inflammatory and anti-immune mechanism in Mets. The optimal benefit of SCFAs in Mets could provide a refreshing opportunity for future prevention and treatment of Mets.
