# Corrigendum: The role of short-chain fatty acid in metabolic syndrome and its complications: focusing on immunity and inflammation

**DOI:** 10.3389/fimmu.2025.1580492

**Published:** 2025-03-07

**Authors:** Wenqian Yu, Siyuan Sun, Yutong Yan, Hong Zhou, Ziyi Liu, Qiang Fu

**Affiliations:** ^1^ Dongzhimen Hospital, Beijing University of Chinese Medicine, Beijing, China; ^2^ First Clinical Medical College, Beijing University of Chinese Medicine, Beijing, China

**Keywords:** short chain fatty acids, metabolic syndrome, inflammation, immunity, complications

In the published article, there was an error in the author list, and author Yutong Yan^1,2^, Hong Zhou^1,2^, Ziyi Liu^1,2^ was erroneously excluded. The corrected author list appears below.

“Wenqian Yu^1,2&^, Siyuan Sun^1,2&^, Yutong Yan^1,2^, Hong Zhou^1,2^, Ziyi Liu^1,2^, Qiang Fu^1^*”

In the published article, there was an error in **Author contributions**. Due to author changes, there are three authors whose contributions need to be added to the **Author Contributions** section. The correct material statement appears below.

“WY: Conceptualization, Software, Writing – original draft, Writing – review & editing. SS: Data curation, Writing – original draft, Writing – review & editing. YY: Data curation, Writing – revised draft, Writing – review & editing. HZ: Data curation, Writing – revised draft, Writing – review & editing. ZL: Data curation, Writing – revised draft, Writing – review & editing. QF: Methodology, Writing – original draft, Writing – review & editing.”

In the published article, there was an error in **Acknowledgments**. **Acknowledgments** were not added to the original article, and since another person contributed to the revision process of this article, a new acknowledgement should be added. The correct material statement appears below.

“We would like to thank Jiaying Yan for her contribution to section 5.2 during the revision of this paper.”

The authors apologize for this error and state that this does not change the scientific conclusions of the article in any way. The original article has been updated.

